# Internet-versus group-administered cognitive behaviour therapy for panic disorder in a psychiatric setting: a randomised trial

**DOI:** 10.1186/1471-244X-10-54

**Published:** 2010-07-02

**Authors:** Jan Bergström, Gerhard Andersson, Brjánn Ljótsson, Christian Rück, Sergej Andréewitch, Andreas Karlsson, Per Carlbring, Erik Andersson, Nils Lindefors

**Affiliations:** 1 Karolinska Institutet, Department of Clinical Neuroscience, Center for Psychiatry Research, Stockholm, Sweden; 2 Linköping University, Department of Behavioural Sciences and Learning, Swedish Institute for Disability Research, Linköping, Sweden; 3 Mid Sweden University, Department of Social Sciences, Section of Psychology, Östersund, Sweden; 4 Umeå University, Department of Psychology, Umeå, Sweden

## Abstract

**Background:**

Internet administered cognitive behaviour therapy (CBT) is a promising new way to deliver psychological treatment, but its effectiveness in regular care settings and in relation to more traditional CBT group treatment has not yet been determined. The primary aim of this study was to compare the effectiveness of Internet-and group administered CBT for panic disorder (with or without agoraphobia) in a randomised trial within a regular psychiatric care setting. The second aim of the study was to establish the cost-effectiveness of these interventions.

**Methods:**

Patients referred for treatment by their physician, or self-referred, were telephone-screened by a psychiatric nurse. Patients fulfilling screening criteria underwent an in-person structured clinical interview carried out by a psychiatrist. A total of 113 consecutive patients were then randomly assigned to 10 weeks of either guided Internet delivered CBT (n = 53) or group CBT (n = 60). After treatment, and at a 6-month follow-up, patients were again assessed by the psychiatrist, blind to treatment condition.

**Results:**

Immediately after randomization 9 patients dropped out, leaving 104 patients who started treatment. Patients in both treatment conditions showed significant improvement on the main outcome measure, the Panic Disorder Severity Scale (PDSS) after treatment. For the Internet treatment the within-group effect size (pre-post) on the PDSS was Cohen's *d *= 1.73, and for the group treatment it was *d *= 1.63. Between group effect sizes were low and treatment effects were maintained at 6-months follow-up. We found no statistically significant differences between the two treatment conditions using a mixed models approach to account for missing data. Group CBT utilised considerably more therapist time than did Internet CBT. Defining effect as proportion of PDSS responders, the cost-effectiveness analysis concerning therapist time showed that Internet treatment had superior cost-effectiveness ratios in relation to group treatment both at post-treatment and follow-up.

**Conclusions:**

This study provides support for the effectiveness of Internet CBT in a psychiatric setting for patients with panic disorder, and suggests that it is equally effective as the more widely used group administered CBT in reducing panic-and agoraphobic symptoms, as well as being more cost effective with respect to therapist time.

**Trial registration:**

ClinicalTrials.gov NCT00845260

## Background

Panic Disorder with or without agoraphobia (PD/A) is a common and, if untreated, usually chronic psychiatric disorder shown to be associated with impaired function and an elevated risk of suicide and premature death [1,2]. Effective pharmacological treatment for PD/A is principally in the form of the selective serotonin reuptake inhibitors (SSRI) [3], whereas the psychological treatment with the clearest evidence base is cognitive behaviour therapy (CBT) [4]. Psychodynamic therapy is another potentially effective psychological treatment [5]. Combining CBT with SSRI does not seem to lead to better treatment response than CBT alone [6].

However, while access to pharmacological treatments can be considered satisfactory in most cases, access to CBT is, in contrast, often limited [7]. This is probably in large part due to a lack of trained therapists, especially outside of specialised health care centres and larger cities. In response to this situation, more accessible CBT treatment formats for PD/A have been developed. Group CBT is probably the most common format used to increase the number of patients getting access to evidence-based psychological treatment. Group CBT for PD/A has been tested in a number of clinical trials [8], and has also been evaluated in a regular care setting [9].

Another way to increase the accessibility of CBT is Internet administered treatment, which stems from research on bibliotherapy [10]. A number of controlled trials have been published showing the efficacy of Internet-based CBT for PD/A [11-15]. However, all of these trials have evaluated the treatment in research settings, with self-recruited participants. Only one small open effectiveness trial has evaluated Internet treatment for PD/A [16].

To our knowledge, Internet-based treatment has not been evaluated in a randomised trial in a regular psychiatric health care setting for any psychiatric disorder. Research designs in regular care settings with the goal of maximising external validity are often called "effectiveness" studies (in contrast to "efficacy") and are considered to be an increasingly important part of clinical research [17]. In such trials patients are preferably referred in a regular manner to treatment and extensive exclusion criteria should not be used. Moreover, those performing treatment should preferably be regular staff not specially trained for participation in the trial and the patients in the trial should not receive more special attention or additional treatment interventions in comparison to what patients normally would receive.

Another aspect of clinical research receiving increasing amount of attention in the literature is cost-effectiveness analysis [18,19]. In the light of the issues of dissemination and accessibility of psychological treatments raised earlier, formal evaluations of the relation between costs of treatment delivery and effects of treatment are crucial.

In the present randomised trial, the aim was firstly to compare Internet-based CBT to group CBT for patients diagnosed with panic disorder in a regular psychiatric setting. We hypothesized that the two treatment formats would both be effective, based on the established efficacy of both group [8] and Internet delivered CBT [20], and two previous efficacy studies comparing live individual and Internet treatment [21,22] which showed no major differences between the two treatment formats.

Secondly, our aim was also to evaluate the cost-effectiveness (concerning therapist time) of Internet-based CBT in relation to the more traditional group CBT, which is currently considered to be the most cost-effective psychological treatment commonly used in clinical settings for PD/A.

## Methods

### Recruitment and selection

Patients were consecutively referred for participation in the study from either psychiatric outpatient clinics or general practitioners. However, a minority of patients (one third) were self-referred to the Anxiety Disorders Unit at the Psychiatric Clinic of Karolinska University Hospital, where the trial was conducted. First, all patients were interviewed by a research nurse in a short telephone screening interview. This interview established the presence of current panic attacks, that the patient consented to be randomised, resided in Stockholm County, and that he or she had daily Internet access.

Those not excluded in the short screening interview were then assessed in an in-person structured clinical interview conducted by a psychiatrist, or a resident in psychiatry under the supervision of a senior psychiatrist. The diagnostic part of the clinical interview was based on the Mini-International Neuropsychiatric Interview (M.I.N.I.) [23]

To be included in the study the patients had to meet the following criteria: 1. Fulfil DSM-IV criteria for panic disorder with or without agoraphobia (PD/A), 2. Have PD/A as primary diagnosis, 3. Be above 18 years of age, 4. Not suffer from severe depression or suicidal ideation, 5. If taking prescribed drugs for panic disorder, having had a constant dosage for 2 months prior to commencing treatment in the study, 6. Not undergoing concurrent CBT.

The study protocol was approved by the Regional Ethical Review Board, Stockholm, Sweden. Written informed consent was obtained from all participants after the procedure had been fully explained by the psychiatrist.

### Materials

All patients were required to have regular daily Internet access as well as the possibility to print text materials used.

### Outcome measures

The primary outcome measure was the clinician rated Panic Disorder Severity Scale (PDSS) [24]. It measures the frequency of full panic attacks as well as limited symptom attacks. It also rates the experienced distress from attacks, worry about attacks, effect of PD on social and professional functioning, as well as degree of interoceptive- and agoraphobic avoidance. Other outcomes measures used were the Clinical Global Impression Scale (CGI) [25], the Montgomery Åsberg Depression Rating Scale (MADRS) [26], the Anxiety Sensitivity Index (ASI) [27], and the Sheehan Disability Scale (SDS) [28]. Information on current work- and/or sick leave-status was obtained in the interview, along with information on duration of PD, history of psychiatric- and/or somatic illness, and current medication.

All outcome measures have previously established adequate psychometric properties and were administered during the clinical interview by a psychiatrist at pre-and post-treatment, as well as after a 6-month follow-up period.

### Response

Treatment response was evaluated in two different ways, taking into account two different clinician rated measures, the PDSS and the CGI [25]. For the PDSS a patient was considered as a responder when a 40% reduction from baseline to post-treatment on the PDSS was observed, as defined in other trials on PD/A [5,29]. For the CGI, a patient was defined as being a responder if considered to be "much improved" or better on the CGI improvement subscale, while being rated as "mild" or less on the CGI severity subscale. The number of participants in remission after treatment was evaluated by calculating the proportion of patients no longer fulfilling DSM-IV PD/A diagnosis at the clinical interview at post-treatment and follow-up.

### Procedure

**Figure 1:**
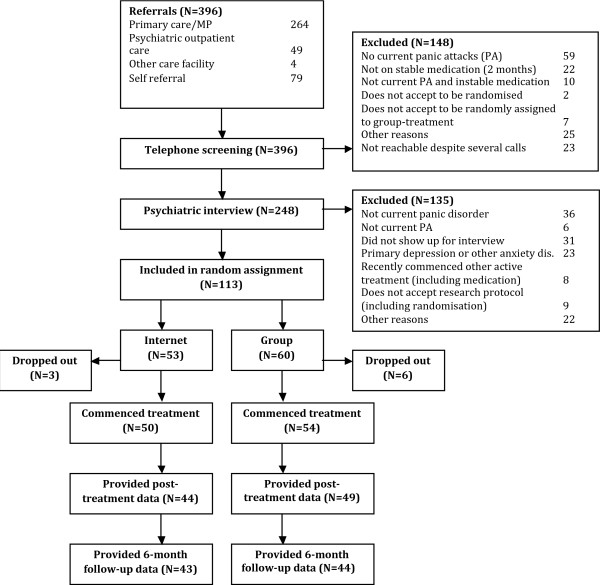
**Flowchart of study participants, point of random assignment, and dropouts**.

An overview of the procedure is given in Figure 1. Patient characteristics are given in Table 1. We aimed to include all types of PD/A patients that normally would receive CBT for panic disorder at our clinical unit. There were no significant differences in these characteristics between the two treatment groups, except for type of referral and type of psychotropic medication. Although the proportion of patients taking any psychotropic medication did not differ between groups, patients randomised to the group treatment were to a larger extent on benzodiazepine derivate or neuroleptic medication, and fewer were on SSRI/SNRI medication, than was the Internet group (see Table 1).

**Table 1:** Characteristics of participants at the start of the trial.

	Internet n = 50	Group n = 54
Age, years: mean (SD)	33.8 (9.7)	34.6 (9.2)
Gender: female, %	64%	59%
Duration of PD, years (SD)	6.0 (9.3)	7.3 (9.6)
Comorbid agoraphobia	86%	83%
On sick leave (part-or fulltime)	20%	26%
Referral: Psychiatric out-patient clinics, %	6%	13%
General practitioners	63%	52%
Self-referrals	31%	35%
Any psychotropic medication	44%	46%
SSRI/SNRI	44%	24%
Benzodiazepine	10%	15%
Benzodiazepine derivate/neuroleptic	14%	33%
Tricyclic antidepressive	4%	5%

The participants were divided into two groups, Internet- or group treatment, by an independent random-number procedure, where each patient was assigned to either treatment by the opening of sealed numbered envelopes. Nine participants dropped out after randomisation but before commencing treatment. Various reasons were given for not starting treatment, but all pertained to different life circumstances of the individual participants and not to randomisation status. These initial dropouts were excluded from the statistical analyses.

A number of patients did not return for the clinical interview at post-treatment or follow-up. As suggested by Gueorguieva et al. [30], a mixed effects models approach was used in the statistical analysis to adjust for these missing values. The psychiatrists performing the clinical interviews at post-treatment and follow-up were blind to treatment condition.

### Internet treatment

The treatment programme consisted of 10 self-help modules which were based on established CBT principles [31]: psychoeducation (module 1), cognitive restructuring (modules 2 and 3), interoceptive exposure (modules 4 and 5), exposure in-vivo (for agoraphobic situations; modules 6 to 9), and relapse prevention (module 10).

In the Internet treatment the self-help programme was administered via web pages. The text modules consisted of information as well as exercises, to be performed in the patient's every-day life. Each module ended with a number of questions to be answered by the patient through interactive forms (e.g. homework assignments). After reviewing these answers, the psychologist gave access to the next module and provided feedback. At any moment the patient could post a message if he or she needed further help. Messages were answered within 24 hours on regular weekdays. No other contact than by e-mail between patient and psychologist took place during the treatment. The patient also had the opportunity to participate in an online discussion forum with other patients in treatment during the same time period. However, this was not mandatory.

### Group treatment

The group treatment was led by two clinical psychologists who presented the self-help programme mentioned above during weekly 2-hour sessions, with the support of printed handouts of the modules given to the patients. The homework assignments described above were addressed during the group sessions. The psychologists involved in the treatment were regular staff psychologists not specially trained for participation in the trial. Both the Internet and the group treatment were 10 weeks long (1 module/group session per week). The patients in the trial did not receive more special attention or additional treatment interventions in comparison to other patients at our clinical unit.

### Statistical analysis and rationale for comparisons

We were informed by the adapted CONSORT checklist [32], but also analysed our data according to a mixed models approach. We begin by presenting the raw scores and mean standardized differences (Cohen's d), based on the pooled standard deviation.

The power for the within-group contrasts were estimated based on a conservative effect size of d = 0.80, and the sample sizes in each group were regarded as sufficient to detect a within-group effect of this size. Given the previous literature on the effects of CBT for panic disorder we considered a mean standardized difference at or below d = 0.20 as the criteria for equivalence for the main outcome measure PDSS. This is in line with previous psychotherapy research in which d = 0.20 is regarded as a minor difference of little clinical importance [33]. We also calculated 95% confidence intervals for the between group effect size. However, we were not able to power the study for the reliable detection of a small between group effect. The obtained power was only robust for a large difference of d = 0.50 (two-tailed test, power 75%), which was well above our criteria of equivalence. We also present response rate in categorical terms in raw percentages. For equivalence regarding proportion of responders a difference of 10% or more on the main outcome measure was regarded as non-equivalence, but again we did not have enough power to detect a small effect.

For the within-group comparisons missing data is critical as effects could be overestimated. As a second way to analyze the data, and to account for missing data we used a mixed effects models approach [30] because in the analysis of longitudinal data repeated observations for the same individual are correlated. This correlation violates the assumption of independence necessary for more traditional, repeated-measures analysis and leads to bias in regression parameters. Typically, ignoring the correlation of observations leads to smaller standard errors (SEs) and increases type I errors, which might lead to the wrong conclusion [34]. Furthermore, mixed effect models are able to accommodate missing data and the integration of time-varying factors, which are issues in the present study. To compare the Internet-based and group treatment according to the outcome measures at baseline, post treatment and 6 months follow-up we used a covariance pattern model [34], which is a special case of mixed-effects models. A separate model was estimated for each of the 8 outcome factors, listed in Table 3. The variance-covariance for each model was assumed to be block diagonal but unstructured within a block defined by subjects. To study if the effect of treatment differed across the time points, we tested the interaction between time and treatment. We used the restricted maximum likelihood (REML) as our model estimation method and present the estimated means and difference between treatments and their respective standard error means (SEs). All these analyses were performed in SPSS version 15.0 (SPSS Inc., Chicago, IL).

Cost-effectiveness ratios were estimated by dividing the treatment cost (of therapist time) with the treatment outcome. In addition, incremental cost-effectiveness was determined using a regression framework with costs and effects as dependent variables (based on 10,000 bootstrap replications). Cost-effectiveness data were analysed using Stata 10.0 S/E (StataCorp Inc.)

All participants who attended at least one Internet- or group session are included in the analysis (n = 104).

## Results

### Effect sizes

Raw means, standard deviations, as well as between- and within group effect sizes based on completer data are presented in Additional file 1. As seen in Additional file 1 the between group effect size for the main outcome measure PDSS was d = 0.00 (CI95% = -0.41 to 0.41) at post-treatment. The between group effect size at 6-month follow-up was d = 0.23 (CI95% = -0.15 to 0.62) for the PDSS.

### Categorical measures and response rate

As shown in Table 2, a majority of patients responded to treatment, when response was defined as a 40% decrease in PDSS scores from pre- to post-treatment and from pre-treatment to follow-up. This was also the case for the CGI and status of PD/A diagnosis. Dropouts (those patients who refused the post-treatment and/or follow-up interview) were regarded as non-responders.

**Table 2:** Proportion of responders and proportion free of PD diagnosis at post-treatment and at follow-up. Dropouts are regarded as non-responders.

Measure	Group	Post	CI 95%	Follow-up	CI 95%
**PDSS**	Internet	60%	46% - 74%	71%	58% - 85%
	Group	63%	50% - 76%	65%	52% - 78%
**CGI**	Internet	50%	36% - 64%	70%	57% - 83%
	Group	59%	46% - 73%	61%	48% - 75%
**PD free**	Internet	60%	46% - 74%	70%	57% - 83%
	Group	63%	50% - 76%	59%	46% - 73%

### Mixed models

In Table 3 we present mean estimates from the mixed effects model and associated *p*-values. As evident from Table 3 the results from the mixed effect models clearly show that both treatments had significant impact on all outcome measures over time. However, there were no interactions or differences in estimated means.

**Table 3:** Results from mixed effects models accounting for missing data.

Measure	Time	Mean Estimates (SE)	*P * (Time)	Treatment difference Internet-group (SE)	*P * (Difference)	*P * Interaction time * treatment
**PDSS**	Pre	14.1 (0.4)	.000	0.0 (0.8)	.99	.46
	Post	6.4 (0.5)		0.1 (1.1)	.95	
	F-U	4.6 (0.5)		-1.0 (1.0)	.34	
**MADRS**	Pre	9.2 (0.5)	.000	-0.6 (1.0)	.52	.98
	Post	4.6 (0.5)		-0.8 (1.0)	.44	
	F-U	4.3 (0.7)		-0.6 (1.3)	.67	
**ASI**	Pre	32.9 (1.2)	.000	-0.7 (2.4)	.77	.36
	Post	17.0 (1.1)		3.0 (2.3)	.19	
	F-U	16.6 (1.2)		1.3 (2.4)	.59	
**SDS 1**.	Pre	5.7 (0.3)	.000	-0.4 (0.6)	.50	.82
	Post	2.6 (0.3)		-0.8 (0.6)	.23	
	F-U	2.2 (0.3)		-0.6 (0.6)	.353	
**SDS 2**.	Pre	5.7 (0.3)	.000	0.4 (0.5)	.48	.65
	Post	2.8 (0.3)		-0.1 (0.6)	.85	
	F-U	1.9 (0.3)		-0.7 (0.6)	.25	
**SDS 3**.	Pre	4.4 (0.3)	.000	0.4 (0.5)	.41	.52
	Post	1.9 (0.3)		-0.9 (0.5)	.09	
	F-U	1.6 (0.3)		-0.3 (0.5)	.53	

### Therapist time and cost-effectiveness

The average number of weekly modules completed in the Internet treatment was 6.7 (SD = 2.5). The total number of e-mails sent by the therapists during treatment was 555 (mean per patient: 11.3, SD = 4.3). The total average therapist time spent per patient in the Internet treatment was 35.4 minutes (SD = 19.0). That is, this was the mean amount of time that therapists used to answer e-mails from each patient. As evident from the standard deviation, there was great variance in individual therapist time, largely reflecting the relatively large variance in modules completed. The total average therapist time spent per patient in the group treatment was 6 hours, considering that the 54 group patients were distributed over 10 different groups whose sessions were 2 hours each and led by 2 therapists, and that the actual average number of weekly group sessions attended in the group treatment was 8.1 (SD = 2.1). Group CBT thus utilised considerably more therapist time than did Internet CBT.

The direct cost of the Internet treatment (therapist time and the cost of psychiatrist evaluation) was on average 86 euros per patient whereas it was 325 euros for the group treatment. We did not calculate overhead costs (such as treatment development costs for website, treatment protocol etc). Defining effect as proportion of PDSS responders, the cost-effectiveness analysis showed that Internet treatment had superior cost-effectiveness ratios in relation to group treatment both at post-treatment and follow-up (see Table 4). The direct cost of treatment for each additional PDSS responder was at post-treatment 516 euros for group treatment and 143 euros for Internet treatment. At follow up, this cost was 500 euros and 121 euros respectively.

**Table 4:** Comparative cost analysis and cost-effectiveness ratios at post-treatment and follow-up.

	Group CBT		Internet CBT	
	**Post treatment**	**Follow-up**	**Post treatment**	**Follow-up**

**Therapist cost**	260		21	
**Psychiatrist cost**	65		65	
**Total costs**	325		86	
**Proportion of PDSS responders**	0.63	0.65	0.60	0.71
**Cost-effectiveness ratio**	516	500	143	121

**Figure 2:**
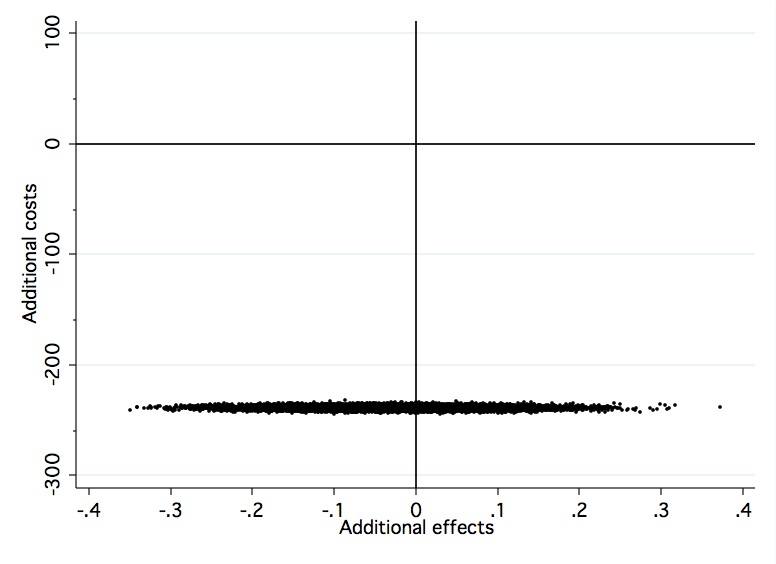
**Cost-effectiveness plane for results at post-treatment**.

**Figure 3:**
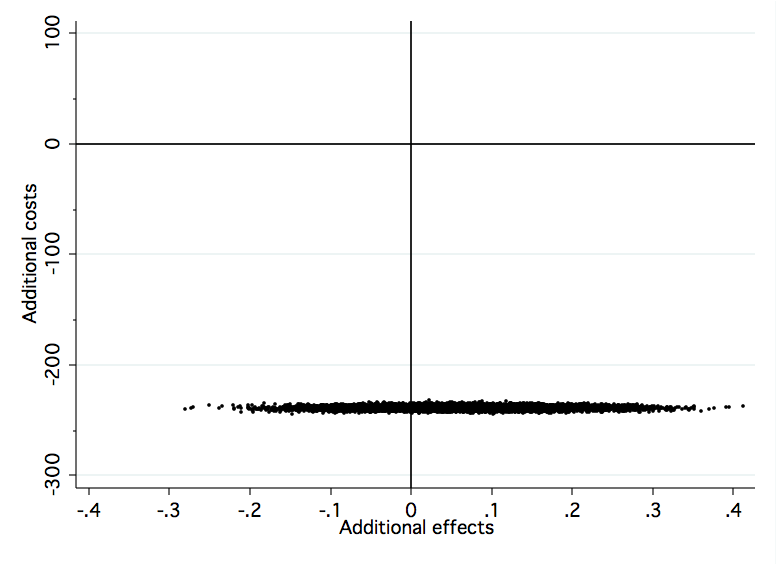
**Cost-effectiveness plane for results at follow-up**.

Figures 2 and 3 are visual presentations of the incremental cost-effectiveness of delivering Internet CBT at post-treatment and follow-up. The x-axis represents the additional effects, that is, dots located to the right ("east") of zero on the x-axis represents the additional effects of offering Internet CBT as opposed to group CBT. The y-axis represents the funding needed to produce such an effect. Dots located below ("south of") zero on the y-axis means that cost savings are generated when offering Internet CBT as opposed to group CBT. As seen in Figure 2, at post treatment, 62% of the dots are located in the south west quadrant indicating that Internet CBT generates slightly lesser effects compared to group CBT but to a cost saving of € 239. As seen in Figure 3, at follow-up, 75% of dots are located in the south east quadrant, indicating that additional effects are achieved alongside cost-savings.

## Discussion

This study provides evidence for the effectiveness of Internet CBT in a psychiatric setting for referred patients with panic disorder, and suggests that it is equally effective as the more widely used group administered CBT. Both treatments showed large within group effect sizes both at post-treatment and at 6-month follow-up on primary as well as secondary outcome measures. In addition, Internet CBT was more cost-effective than group CBT with respect to direct costs in terms of therapist time.

The treatment effects found in the trial are comparable to those found in other trials of both pharmacological and psychological treatments [29]. More specifically, panic severity was significantly reduced (frequency and distress of panic attacks, as well as agoraphobic avoidance). Depressive symptoms were equally reduced in both groups, as well as anxiety sensitivity. Furthermore, after treatment patients reported less disability both in work-, social- and family life. Within-group effect sizes were in line with previous studies on CBT for panic disorder [4].

A majority of patients were considered as responders to treatment, both when this was defined as a significant drop in panic symptoms as well as when defined as degree of global improvement and end-state functioning. Moreover, a majority of patients no longer fulfilled DSM-IV criteria of panic disorder after treatment, and this proportion of patients increased somewhat at the 6-month follow-up.

Given low statistical power for detecting a reliable difference between the two treatments, equivalence between Internet and group CBT for panic disorder cannot be confidently established. However, overall the data suggests that more than half in each group responded to treatment with a substantial decrease in symptoms. This is in line with Barlow and co-workers who had a somewhat lower percentage of responders [29], but slightly lower than Milrod et al. who had a higher percentage of responders [5].

Because we did not include an untreated control condition, the effect of spontaneous improvement was not controlled for. However, in earlier trials where such control conditions have been included, they have not showed significant improvement in symptom severity [35]. In addition, our aim was not to show that Internet-delivered CBT is better than just being on a waiting list as this has been established previously [11,13].

The amount of treatment completed within the 10-week time frame was slightly lower in the Internet treatment than in the group treatment (6.7 modules versus 8.1 group sessions completed). This did not however seem to influence treatment outcome, nor did the fact that patients in the group treatment received considerably more therapist attention.

The cost-effectiveness analysis showed that Internet treatment had superior cost-effectiveness ratios in relation to group treatment both at post-treatment and follow-up concerning direct costs of therapist time and psychiatrist assessment. Therapist time, being the only varying factor of the two, is the one of primary interest. However, no formal analysis was made of indirect overhead costs related to development of treatment manuals, website development, and other facilities at the clinical unit where the treatments were developed and conducted. Therefore the conclusions that can be drawn from the cost-effectiveness analysis are limited, and are restricted solely to therapist time. However, given that only the group treatment uses the traditional facilities at the clinical unit such as its premises, reception etc, including such costs could be even more detrimental to the cost-effectiveness of this treatment format.

In the present paper we did not focus on predictors of outcome or mediators of the results. For this additional data analyses will be required.

To our knowledge this was the first study comparing Internet administered CBT with group CBT with referred patients in a regular psychiatric setting, for any psychiatric disorder. We argue that Internet-delivered CBT could be a suitable way of disseminating evidence-based psychological treatment, at least as a complement to existing treatment. Internet is an increasingly accessible medium all over the world. For example, in Sweden 89.2% of the population is estimated to have Internet access [36]. Internet-delivered CBT allows the individual patient to engage in treatment and to be guided by a CBT therapist without having to accommodate to office appointments. Web-based applications allows for the use of interactive forms and questionnaires with several advantages over pen-and-paper forms used in traditional CBT, both by aiding the individual patient in doing exercises and in monitoring his or her progress, and by allowing the therapist to have instant access to data during treatment. The literature [37] strongly suggests that guidance/therapist contact during treatment is needed, as non-guided Internet treatments generally show smaller or nonexistent treatment effects and much larger attrition. In one evaluation of an open access web-based CBT programme (with neither stringent diagnostic procedure nor therapist guidance), only 1% of registered users completed treatment [38]. In our treatment each individual patient was assessed in a diagnostic interview by a psychiatrist as well as guided through treatment by an individual therapist. This is assumed to account for the robust treatment effect and relatively low attrition rate. However, the role of therapist guidance, and more specifically the sufficient amount of therapist time or degree of therapist engagement, should be directly evaluated within this treatment setting.

## Conclusions

The results from this trial provide support for the use and dissemination of Internet-based treatment for panic disorder within psychiatry. Our findings suggest that Internet CBT is an effective treatment in this setting and that it is considerably more cost-effective than the more commonly used group CBT. Internet treatment, being a novel treatment approach, has the potential to greatly increase access to evidence based psychological treatments within the health care system.

## Competing interests

The authors declare that they have no competing interests.

## Authors' contributions

JB conceived of the study and its design, was the project manager, participated in the drafting of treatment manuals, performed treatments, and participated in analysis and interpretation of data as well as drafted the manuscript. GA participated in the conception of the study and its design, in analysis and interpretation of data, performed statistical analysis, and participated in the drafting of the manuscript. BL participated in project management, performing of treatments and data analysis as well as in the revision of the manuscript. CR participated in the conception of the study and its design, performed psychiatric interviews and assessment as well as participated in the revision of the manuscript. SA participated in the conception of the study and its design, performed psychiatric interviews and assessment as well as participated in the revision of the manuscript. AK participated in the conception of the study and its design, in the drafting of treatment manuals as well as in the revision of the manuscript. PC participated in the drafting of treatment manuals, in analysis and interpretation of data, performed statistical analysis, as well as participated in the drafting of the manuscript. EA performed cost-effectiveness analyses, and participated in the revision of the manuscript. NL participated in the study conception, its design and management, analysis of data, interpretation and drafting of the manuscript. All authors read and approved the final manuscript.

## Pre-publication history

The pre-publication history for this paper can be accessed here:

http://www.biomedcentral.com/1471-244X/10/54/prepub

## Supplementary Material

Additional file 1 **Means (SD) for the continuous scales used at pre-, post and follow-up, as well as between- and within group effect sizes (Cohen's d)**. PDSS: Panic Disorder Severity Scale. MADRS: Montgomery Åsberg Depression Rating Scale. ASI: Anxiety Sensitivity Index. SDS: Sheehan Disability Scale. Click here for file
