# Supplementary material for: Internet-versus group-administered cognitive behaviour therapy for panic disorder in a psychiatric setting: a randomised trial
Source: BMC Psychiatry. 2010 Jul 2;10:54. doi: 10.1186/1471-244X-10-54 (PMC2910662; doi:10.1186/1471-244X-10-54)
Supplement: Additional file 1 — Means (SD) for the continuous scales used at pre-, post and follow-up, as well as between- and within group effect sizes (Cohen's d). PDSS: Panic Disorder Severity Scale. MADRS: Montgomery Åsberg Depression Rating Scale. ASI: Anxiety Sensitivity Index. SDS: Sheehan Disability Scale. [file 1471-244X-10-54-S1.PDF]

Measure	Group	Pre	Post	Effect size (between)	Effect size (within) Pre-Post	6-month Follow-up (FU)	Effect size (within) Pre-FU
**PDSS**	Internet	14.1 (4.3)	6.3 (4.7)	0.00	1.73	4.1 (4.2)	2.35
	Group	14.2 (4.0)	6.3 (5.6)		1.62	5.0 (5.3)	1.96
**MADRS**	Internet	8.9 (5.2)	4.1 (3.8)	0.20	1.05	4.1 (6.1)	0.85
	Group	9.5 (4.9)	5.1 (5.7)		0.83	4.9 (6.6)	0.79
**ASI**	Internet	32.5 (11.6)	18.1 (12.1)	0.24	1.21	16.6 (11.9)	1.35
	Group	33.2 (12.4)	15.4 (10.0)		1.58	16.1 (11.1)	1.45
**SDS 1. Work**	Internet	5.5 (3.1)	2.2 (2.6)	0.30	1.15	1.9 (2.8)	1.22
	Group	5.9 (2.8)	3.1 (3.3)		0.92	2.6 (3.3)	1.08
**SDS 2. Social**	Internet	5.6 (2.8)	2.6 (2.8)	0.14	1.07	1.6 (2.5)	1.51
	Group	5.9 (2.5)	3.0 (3.0)		1.06	2.3 (3.0)	1.30
**SDS 3. Family**	Internet	4.1 (2.9)	1.4 (1.9)	0.41	1.10	1.5 (2.1)	1.03
	Group	4.6 (2.5)	2.4 (2.8)		0.83	1.8 (2.7)	1.08
